# Notes and News

**Published:** 1889-10-19

**Authors:** 


### Notes and News.

Collected and Communicated.

Sidmouth is not going to let slip the generous offer of £500 towards establishing a cottage hospital in that town. At a special meeting of the committee held on October 9th, the scheme was warmly commended, and another £500 was promised by those present.

Things are not working quite smoothly at the Worcester Dispensary and Provident Medical Institution ; the medical gentlemen are only paid at the rate of 3d. a visit, yet some new rules are proposed which will place the remuneration of the medical officers entirely in the hands of the committee. The medical officers fear the committee may indulge in new buildings, etc., and leave the officers without any pay at all. Finally a committee containing three medical men was elected to consider the proposed new rules.

The opening of the medical session of Owens College was celebrated by a conversazione. Dr. Young, Dean of the Medical School, read the annual report of the department of medicine. It stated that the total number of students attending the department during the session 1888-9 was 381, and furnished in detail the list of scholarships, exhibitions, and prizes awarded during the session. The increase in the number of students for the year was the largest which had taken place since the medical department was instituted, the number (381) showing an increase of 48 over that for the previous year.

Seeing the great desirability of extending the present buildings of the Boscombe Hospital, the committee, at a recent meeting, unanimously agreed to adopt plans submitted to them providing for a new surgery and consulting-room, additional bedrooms for the staff, lavatories, and, in particular, an accident ward—a special need —besides other improvements. The sum of £400 required for these items will, it is earnestly hoped, be ungrudgingly supplied by those who are acquainted with or may have occasion to be grateful to the health-giving breezes—laden with pine odours—of Bournemouth's prettiest suburb.

Dr. Arlidge has been reading a paper before the Manchester Statistical Society on the causes of diseases in manufacturing pursuits. Dr. Arlidge has gone to Manchester on purpose to study this subject, and his conclusions so far point to the following as the principal causes of illness amongst workers in manufactories : dust, poisonous materials, metallic and non-metallic ; noxious vapours, animal, vegetable, and mineral ; excessive temperatures, heat and cold ; electricity, atmospheric pressure, increased and decreased ; conditions affecting the special senses, overuse and overstrain of parts of the body, exposure to infectious or contagious diseases or to parasites, occupation fraught with accidents, and sedentary employment.

The Secretary of the Charity Reform Voting System has written to the *Standard* vindicating the society, which, he says, has been severely criticised for abusing the voting system. We hoped the majority of people were entirely in favour of the non-voting system, which certainly commends itself to those imbued with common-sense. We expect to shortly see the total abolition of all voting and canvassing in regard to charities.

A delightful story comes from Leicester of a total abstinence lecturer who was insisting on the deadly and poisonous qualities of alcohol, which he declared to be injurious to all forms of life. In illustration of this theory he threw on a screen a magnified image of a drop of water swarming with animalculæ all wriggling in the liveliest manner, then he added a minute quantity of alcohol to the water, upon which the poor animalculæ all curled up and died. "Lor ! lor !" said an old man in the audience, "be 'e sure, mister, I shan't niver drink a drop of water after seeing that, unless I qualifies it with lots o' speerits to kill the beasties."

The twentieth annual meeting of subscribers to the West of Scotland Convalescent Homes has been held. In the directors' report it was stated that of the total number of convalescents who had passed through the homes in the course of the year, 2,947 was restored to health, and enabled to resume their occupations ; 272 were relieved—that was, they derived benefit from their residence at the homes, though not so well as to resume their work ; 94 did not improve ; four died ; while 173 still remained under treatment. The total income from all sources had been £5,513 1s. 1d. The total expenditure (ordinary) for the year has been £4,323 8s. 1d. In addition there had been expended in the erection of a new wing, in furniture and furnishings, and in various alterations and repairs, £943 3s. 4d. A balance remained in bank of £246.

On the 7th of October an interesting function took place at the Queen's Hospital, Birmingham, when the subscribers to the Special Fund were invited to view the extensions and improvements on which some of the fund has been spent. During former extensions of the wards, the offices had become central, but they are now placed at the end of each wing, and as a result about 20 feet has been added to the length of each ward. A nurses' home, with 26 bedrooms and a common sitting-room, has also been built, and improvements have been effected in the kitchens and cooking apparatus. £7,650 was the actual response to the appeal of the committee, of which £5,500 has been applied to structural improvements, the surplus being devoted to the working capital. All the alterations suggested in the committee's circular letter have been carried out, except the reflooring of the wards, which was not taken in hand as the estimated cost of £1,000 was too heavy for the slender finances of the hospital. In the afternoon a special meeting of the governors was held, at which Sir James Sawyer was elected consulting physician to the hospital, and presented with a testimonial on vacating the post of senior physician. The Rev. J. C. Blissard, who presided, eulogised Sir James Sawyer's active work in connection with the hospital, and in making the presentation expressed the hope that he would have a long and prosperous career. Sir James Sawyer, in reply, reviewed his career since he entered the hospital as a student, and declared his intention of making the office of consulting physician a real one as far as he could fill it. From first to last, he said, he was a doctor, and his truest happiness lay in practising his profession. He accepted the appointment with gratitude, and prayed that the hospital might continue to be a great hospital and a noble medical school, and a source of help and restoration to thousands.

The following vacancies are announced this week, the amount of the salary in each case being given after the name of the institution:

**Resident Medical Officers.**

St. Mary's Hospital, Manchester. House Surgeon.—£120, board, &c.
St. Mary's Hospital, Manchester. Out-Patient Physician.—£70, board, &c.
Liverpool Children's Infirmary. House Surgeon.—£85, board, &c.
North-West London Hospital. Senior Medical Officer.
Burton-on-Trent Infirmary. House Surgeon.—£130.
Wolverhampton Hospital. House Surgeon.—£100, board, &c.
Carlisle Asylum. Junior Medical Officer.—£80, board, &c.
Carmarthen Asylum. Junior Medical Officer. — £105, board, &c.
Liverpool Dispensaries. Two Assistant Surgeons.—£80, board, &c.
Bradford Infirmary. House Physician.—£100, board, &c.
Hospital for Children, Great Ormond-street. House Surgeon.—£50, board, &c.
Sheffield Infirmary. House Surgeon.—£120, board, &c.
Sheffield Infirmary, Assistant House Surgeon.—£80, board, &c.
Stanhope-street Dispensary. Medical Officer. — £105, rooms, &c.
Flintshire Dispensary. House Surgeon.—£100, rooms, &c.

**Non-Resident Medical Officers.**

Birmingham General Hospital. Assistant Physician.—£100.
Owens College, Manchester. Junior Demonstrator of Physiology.—£100.
Liverpool Royal Infirmary. Honorary Surgeon.
Parish of Eday, Orkney. Medical Officer.—£70.

At the recent examination for Entrance Scholarships in the London Hospital Medical College, the first Science Scholarship (£60) was awarded to Mr. C. P. Harris, the second (£40) to Mr. G. H. Cowen. The first Buxton Scholarship (£30) was awarded to Mr. O. O. Williams, and the second (£20) to Mr. E. F. G. Tucker.

The London Fever Hospital is issuing an appeal for subscriptions, and considering the good work it does the funds should be forthcoming. Scarlet fever is epidemic in our midst once more, and a severe strain is likely to be put on all fever hospitals. During last week there were 190 fever admissions into the London hospitals: 27 on Monday, 31 on Tuesday, 31 on Wednesday, 27 on Thursday, 41 on Friday, and 33 on Saturday. The only way to arrest the epidemic is by the isolation offered by special hospitals.

A hospital secretary who has been all round the world thus describes a New York hospital, which he considers the best he has seen: "It is fairly central in the city and is five storeys high. It has a fine, but not an ornamental, façade. All the money has been spent within. Every ward has curved ends, and the junction of every wall with ceiling and floor is a very slight curve. There is not an angle in the building. The floors are all beautifully tiled. The ventilation is wonderful. Beneath every bed is an inverted funnel attached to a pipe, which leads up to a fan above the roof, by means of which exhaust to any extent can be managed. There is a perfect system of heating by hot-air apparatus. All the gasfittings are of nickel, shining like silver. Luxurious chairs are everywhere, and there is a dainty little Turkish carpet beside every bed. Then all sanitary arrangements are perfect, and, strange to say, after three years of investigation amongst all the latest inventions of the world, common plaster well trowelled has been found the best coating for the walls. The common plaster is beautifully painted. Everything is artistic and nice, pot plants standing in all the wards, and on the topmost storey a winter garden, with aviaries and little menageries about." We should consider this too luxurious in England, and find some difficulty in getting rid of patients once admitted.

The residential college at Guy's is growing rapidly, and is watched with interest by numerous students. Guy's is always going in for improvements. The old clinical wards—which used to be lunatic wards a hundred years ago—have been vastly improved, and will shortly be opened. All drains have been removed from their old position under the building, a new kitchen has been fitted up with a range, and Mr. Bridgin Teale's grates have been introduced into the two wards.

We are all aware that Frenchwomen are particularly subject to *les nerfs*, but the French Academy of Sciences has also discovered this malady in animals. Hysterical phenomena have been remarked in dogs, especially in pet dogs, nursed in the lap of luxury, and deprived of what may be said to constitute a healthy existence for a dog. Many of these pampered four-footed favourites have succumbed, it appears, to nervous disorders, or to violent hysterical attacks, and an examination of their remains after death has proved that the seat of the disorder which cut short their days was merely the nerves. Hysterical women are bad enough, but when it comes to hysterical lap dogs—! Words fail us.

Last week we gave some facts about the different sensibility between men and animals, and now we hear about a paper, lately read by Mr. R. W. Felkin, upon the differences of sensibility between Europeans and negroes. He said it was generally supposed that negroes felt pain to a less degree than Europeans. He found upon testing tactile sensation that the minimum distance at which the two points of the compass could be distinguished at the tip of the tongue was on the average 1·1mm. Europeans, 3mm. in negroes, and 2·6mm. in Soudanese. The tactile sensation of two negro boys who were educated for four years in England became so much more acute that they could distinguish the points of the compass at 2mm. Our boasted civilisation then gives us increased powers of pain as well as increased comforts. Thus is the balance of suffering maintained, and no man allowed to escape his allotted dole of pain.

The death of Dr. James Precott Joule on October 11 removes one of our most eminent authorities on medical electricity. At the meeting of the British Association in Manchester in 1842, Dr. Joule read a paper on the "Electric Origin of Heat," which evoked considerable attention from the savants present, and in the following year, when the association met at Cork, he read a second paper on "The Calorific Effects of Magneto-Electricity and the Mechanical Value of Heat," in which he described a series of experiments in magnetic electricity, made with a view to determine the mechanical value of heat. In an elaborate paper read before the Royal Society in 1849, and published in the "Philosophical Transactions," the results of his experiments are thus stated: "The quantity of heat produced by the friction of bodies, whether solid or liquid, is always proportional to the quantity of force expended;" and secondly, "The quantity of heat capable of increasing the temperature of a pound of water by one degree Fahr., requires for its evolution the expenditure of a mechanical force required by the fall of 772lb. through the space of 1ft." Prior to the successful issue of Mr. Joule's researches, the true principle of the dynamical theory of heat was not experimentally demonstrated. In 1872 he was elected president of the British Association, which in that year held its meeting at Bradford, but before the meeting his health broke down, and he was unable to attend and deliver the customary inaugural address. Since that date he has been almost entirely an invalid, quietly pursuing his studies as far as his health permitted.

